# Uncovering Microbial Diversity and Community Structure of Black Spots Residing in Tomb Mural Painting

**DOI:** 10.3390/microorganisms13040755

**Published:** 2025-03-26

**Authors:** Qiang Li, Zhang He, Zeng Wang, Aidong Chen, Chao Wu

**Affiliations:** 1 School of Art and Archaeology, Zhejiang University, Hangzhou 310028, China; 2 Shaanxi Provincial Institute of Archaeology, Xi’an 710054, China; 3 School of Humanities, Zhejiang University, Hangzhou 310058, China

**Keywords:** tomb mural painting, black spots, *Gliomastix*, co-occurrence patterns, functional predictions

## Abstract

Microbes colonizing cultural artifacts are a ubiquitous phenomenon which may occur during burial, post-excavation, and storage periods, thereby seriously affecting sustainable heritage conservation. In this study, high-throughput sequencing technology was applied to analyze the microbial community structure in ancient mural paintings and the surrounding air, as well as to identify the most characteristic taxa causing black spot contamination. The results showed that members of the genera *Gliomastix* and *Ochroconis* were highly abundant in black-spots-contaminated areas and rarely detected in the air and uncontaminated mural paintings. Air samples of the two tombs showed no significant difference in Chao1 and Shannon indices, whereas statistically significant differences were observed compared to those samples collected from black spots. The taxonomic diversity of the microbial community in the soil-covered mural paintings and air exhibited similar structures at the genus level. Moreover, when compared to other areas of the two tombs, the samples from black spots differed not only in microbial community composition but also in microbial assembly processes and the co-occurrence patterns, such as much less network complexity in the black spots area. Functional predictions uncover the presence of microbial functional profiles involved in nitrogen cycling, organic matter degradation, and animal and human pathogens, representing a potential threat to cultural relics and public health. These results advance our understanding of the impacts of archeological excavations on the microbial community variation in tomb mural paintings.

## 1. Introduction

Microbial colonization on ancient paintings occurs naturally and may cause visible and unforeseen damage to these historically significant cultural artifacts. A number of factors, including storage location, ambient climatic conditions, and pigment composition, have been shown to differentially affect the microbiota composition and structure, thereby inducing different forms of deterioration [1,2,3]. Among these, Actinomycetes are usually considered as pioneer colonizers during a microbial succession and have been widely detected in subterranean ecosystems, such as crypts, tombs, and grottoes [4,5]. Some representative genera of Actinomycetes, including *Streptomyces*, *Nocardia*, *Crossiella*, and *Pseudonocardia*, appear to be adapted to oligotrophic environments and produce novel bioactive compounds with antimicrobial properties [6,7,8]. Phototrophic Cyanobacteria and Chlorophyta are usually detected near the entrances or well-lit areas in subterranean niches, playing a major ecological role in carbon and nutrient cycling through photosynthesis [9]. The occurrence of these phototrophic populations not only decreases the esthetic value of painted artworks by forming characteristic biofilms and incrustations [10], but also contribute to the dissolution and reprecipitation of calcareous rock substrata and may even result in the detachment of parts of the rock and pigment layers [11]. Fungi act as another group of metabolically active biodeteriogens, growing and thriving on a wide range of painted art objects, including canvas paintings [12,13], wall and mural paintings [14,15], and traditional Chinese painting on silk [16]. As far as fungi are concerned, species of *Aspergillus*, *Penicillium*, *Cladosporium*, and *Alternaria* are frequently detected, deteriorating art paintings in a range of forms, such as the secretion of pigments and other coloring agents, the dissolution of mineral components of the plaster [17], and the production of extracellular enzymes and organic acids [18]. Other types of microbial groups, including the sulfate-reducing bacteria [19] heterotrophic *Bacillus* [20,21] and halophilic bacteria [22], have also been found and contribute to discoloration, cracking, salt crystallization, and the degradation of painted artworks.

Regardless of whether it is driven by natural forces or human activities, microclimate fluctuations can directly or indirectly damage paintings by altering microbial community structure and metabolism processes [23,24]. A range of key environmental factors, such as temperature, moisture, ultraviolet radiation, and certain trace gases, affect the initial stages of the ecosystem, either individually or collectively, and alter microbial communities to adapt to the new conditions. For instance, in enclosed or semi-enclosed ecosystems, the emergence and influx of visitors can contribute to atmospheric CO_2_ and organic nutrients levels increase; they can also bring allochthonous microbial populations, thereby disturbing the initial microbial community and ecological equilibrium [25]. The use of light systems can provide visitors with real and pleasant color perception further realizing the value of paintings, but the lighting environment may bring about a variety of adverse effects on high-light-sensitivity artworks, like paintings in museum [26]. By absorbing photon energy from different illumination conditions, paintings exhibit significant differences in deterioration behavior, including those more commonly studied such as discoloration, fading, binders aging, etc. [27,28]. Meanwhile, the installation of artificial lighting in caves and other subsurface environments can lead to the colonization and proliferation of phototrophic microorganisms [29,30]. Generally speaking, cave and tomb ecosystems maintain a constant environmental stress factor [31], including high relative humidity and low air temperature. Once they are discovered or open to large numbers of visitors, spatial-temporal changes in temperature and humidity disturb the distribution of the microbial community structure and even cause an outbreak of specific microbial groups associated with painting deterioration [32]. To inhibit further colonization and the dispersal of indigenous and exogenous microorganisms dwelling on painted artworks, a large amount of research has been carried out, particularly in regard to microbial diversity [33], biodeterioration mechanisms, and preventive and control strategies [34,35].

**Figure 1:**
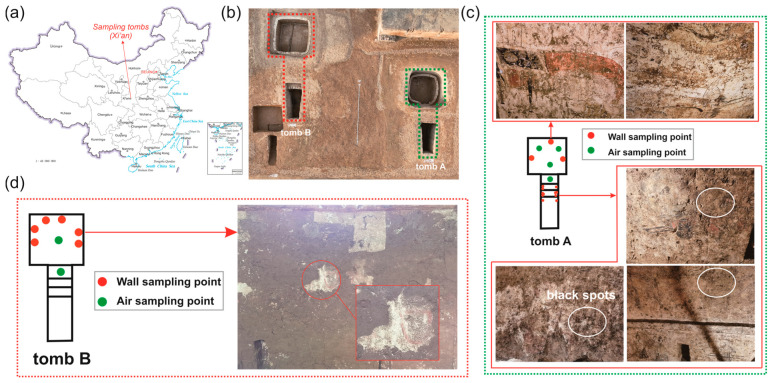
The location of the study site and black spots with signs of fungal colonization on tomb mural paintings. (**a**) The geographical location of two tombs in China, (**b**) view of the tombs, (**c**) wall and air sampling points inside tomb A. The white circle indicates black spots with signs of fungal colonization. (**d**) Wall and air sampling points inside tomb B. The wall sampling sites in tomb B indicate the mural painting areas where the surface soil layers have just been removed.

The aim of this study is to analyze the microbial community composition and understand the role of predominant microbial populations in the deterioration of mural paintings inside the brick tombs in the Xi’an area, Shaanxi province, China. We study two subterranean ancient tombs from the late period of the Tang dynasty (756–907A.D), whose brick wall lime layers were painted with substantial and exquisite patterns. During the excavation of a tomb, we found that when the surface soil was removed, many black spots with features resembling those of fungal colonization appeared on the surface of paintings but not on those areas covered by soil (Figure 1). Therefore, to make clear the cause of the black spots as well as their potential role in mural painting damage, we utilize high-throughput bacterial 16S rRNA genes and fungal internal transcribed spacer (ITS) region sequencing to analyze the microbial community and predict their metabolic pathways. Our work offers valuable insights into problems associated with the microbial contamination of exposed archeological remains, shedding light on the preservative protection of mural paintings in long-term storage.

## 2. Materials and Methods

### 2.1. Site Description and Sampling

The two tombs are 5 m below ground level and consist of one chamber and one corridor, respectively. After being excavated, the gas exchange between the tomb and outdoor environment occurs continuously via an open-air chamber and entrance. Since the surface soil was removed, black spots appeared on both sides of the corridor wall of tomb A, accompanying traces of flowing water, but these phenomena were not observed in chamber and tomb B. A total of 20 samples on mural paintings and 6 indoor air samples were taken (Figure S1). In detail, group AWB includes 9 samples collected from black spots of tomb A; group AWN includes 5 samples collected from the area without macroscopic contamination of tomb A; group AA includes 4 samples collected from air of tomb A; groups BA and BW include 2 air samples and 6 mural painting samples from tomb B, respectively. All mural painting samples were collected using sterilized cotton swabs, while a high-flow bioaerosol sampler (HighBioTrap, Dingblue Tech Inc., Beijing, China) was used for the air sampling at a flow rate of 1000 L/min. The air sampling duration was 15 min for each sample to allow the airborne microbes to settle down on the aluminum foil coating with 600 μL of sterile mineral oil [36]. Sampling was conducted at a height of 1.0 m. All samples were transported to the laboratory on ice for subsequent analysis.

### 2.2. DNA Extraction and Sequencing

Air samples were eluted using a 5 mL 0.05% Tween-20 solution and then filtered through 0.22 μm membranes (Millipore, Bedford, MA, USA) to capture microbial cells. The filtered membranes and cotton swabs samples were used to extract DNA using Qiagen’s DNeasy PowerSoil Pro Kit according to the instructions of the manufacturer. Microbial community composition was analyzed using high-throughput sequencing of the V3-V4 region of the bacterial 16S rRNA gene and ITS1 region of the fungal ITS rDNA gene. PCR primers targeting the 16S rRNA gene sequences were as follows: F (5′-ACTCCTACGGGAGGCAGCA-3′) and R (5′-GGACTACHVGGGTWTCTAAT-3′). PCR primers targeting the fungal ITS region were as follows: F (5′-CTTGGTCATTTAGAGGAAGTAA-3′) and R (5′-GCTGCGTTCTTCATCGATGC-3′). Sequencing was performed on the Illumina NovaSeq 6000 platform of Biomarker Technology Co., Ltd. (Beijing, China). For each sample, PE reads were generated after the filtration of raw data using Trimmomatic (v0.33) and removal of the primer sequence by Cutadapt (v1.9.1). The obtained PE reads were assembled by Usearch (v10.0) and followed by chimera removal using UCHIME (v8.1). DADA2 method in QIIME2 (v2020.6) was applied to the resulting sequences, generating Amplicon Sequence Variants (ASVs). ASVs were taxonomically classified using the Silva database (v138) for bacteria and UNITE database (v8.0) for fungi. All Illumina NovaSeq sequencing data in this study were submitted to the National Center for Biotechnology Information (NCBI) under the Bioproject number: PRJNA1238801 and PRJNA1238803.

### 2.3. Bioinformatic and Statistical Analyses

The alpha diversity of bacterial and fungal community was assessed using the species richness and Shannon diversity index. Microbial community β-diversity was analyzed using Bray–Curtis distance metrics and visualization was achieved via non-metric multidimensional scale analysis (NMDS). The top 80 high-abundance ASVs were selected to build a phylogenetic tree using Qiime relatedness command, while the ggtreeExtra package in R was used to visualize heterogeneous data. The community assembly processes were estimated using null model analysis, which was implemented by the picante (v1.8.2) package in R. Beta nearest taxon index (βNTI) and Raup-Crick (RC_bray_) index were used to assess the variations in phylogenetic and taxonomic diversity [37]. βNTI values smaller than −2 or larger than 2 indicate the dominance of deterministic processes, whereas values within the range of −2 to 2 suggest that stochastic processes play a more important role. Moreover, RC_bray_ values were used as a complement to divide the stochastic processes. RC_bray_ < −0.95 and RC_bray_ > 0.95 indicate homogenizing dispersal and dispersal limitation, respectively, and RC_bray_ values within the range of −0.95 to 0.95 indicate a significant role of undominated assembly. Bacteria, fungi, and inter-kingdom association networks were constructed based on the relative abundances of ASVs and conducted using the ggClusterNet package. Only ASVs with mean relative abundance >0.01% and present in at least one-third of the samples were retained to minimize false-positive signals; then, we set Spearman’s correlation coefficient r to 0.8 and the correlations with a significant *p* < 0.05. Network analysis and calculation of topology parameters were performed using the igraph and microeco [38] package, while co-occurrence networks were visualized in Gephi (v0.10.1). A Zi-Pi plot based on the within-module connectivity (Zi) and among-module connectivity (Pi) values was used to determine the keystone taxa [39]. Functional predictions for bacterial community were performed using the Functional Annotation of Prokaryotic Taxa (FAPROTAX, v1.2.6) and BugBase (0.1.0) tools, while FUNGuild (v1.0) software was used to predict fungal functional groups.

### 2.4. Data of Microclimate Parameters

Environmental parameters, including temperature, humidity, illuminance level, and CO_2_ concentration, were collected using instrument testo 440 with CO_2_ and Lux probes. Before data recording, data logger was placed near the sampling sites for a while to avoid significant fluctuations. The values of environmental parameters corresponding to the sampling site were used for further analysis (Table S1).

## 3. Results

### 3.1. Microbial Diversity Patterns

**Figure 2:**
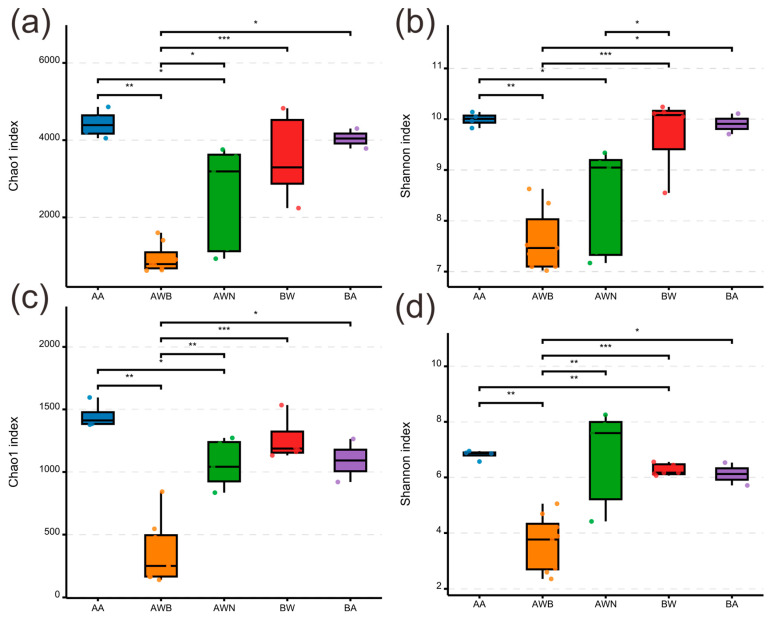
Alpha diversity of bacterial (**a**,**b**) and fungal (**c**,**d**) communities. * *p* < 0.05, ** *p* < 0.01, and *** *p* < 0.001.

**Figure 3:**
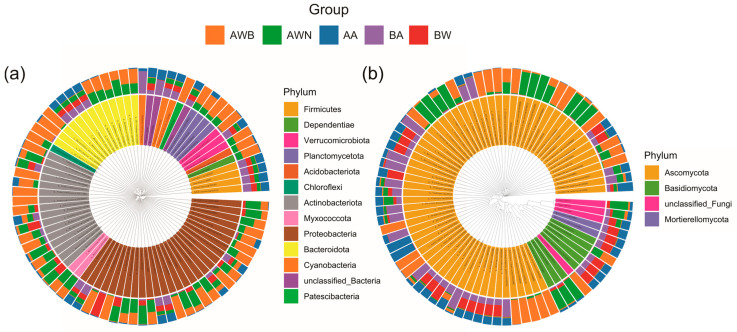
Circular phylogenetic tree uncovering distribution of bacterial (**a**) and fungal (**b**) top 80 high-abundance ASVs in mural painting and air.

**Figure 4:**
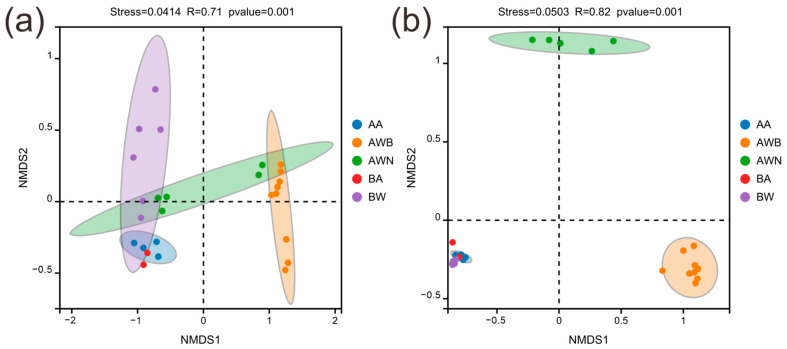
Non-metric multidimensional scale analysis (NMDS) based on Bray–Curtis distance of bacterial (**a**) and fungal (**b**) communities.

For the bacterial communities (Figure 2a,b), group AWB exhibited the lowest Chao1 and Shannon indices, while airborne samples showed a significantly higher species richness and diversity. Species richness levels in groups AWN and BW were relatively consistent, but there were differences in the Shannon index. No significant differences were observed in the Chao1 and Shannon indices between groups BW and BA. The fungal Chao1 richness and Shannon diversity was significantly lower than those in the bacterial community (Figure 2c,d). The samples in groups AA and BA, which were collected from the air in tomb A and tomb B, respectively, showed no significant difference in Chao1 and Shannon indices. In all groups, the most prevalent bacterial phyla were Proteobacteria, Bacteroidota, and Actinobacteriota, accounting for 16.8–45.6%, 7.0–23.3%, and 5.4–24.4%, respectively (Figure S2a). The abundance of these three phyla in group AWB increased compared with group AWN, while the abundance of Firmicutes and Acidobacteriota decreased. At the level of the genus, *Bacteroides*, *Alistipes*, *Pseudonocardia*, and *Akkermansia* were the dominant genera in the bacterial communities, with an average relative abundance of 3.5%, 2.2%, 1.8%, and 1.6%, respectively (Figure S2b). The bacterial composition and abundance exhibited no significant differences between groups AA and BA (Figure 3a), despite the fact that the two groups were collected from different tombs. The dominant phylum of fungal communities was Ascomycota and Basidiomycota, accounting for 54.4–99.5% and 0.2–28.9%, respectively (Figure S3a). In group AWB, the relative abundance of Ascomycota was significantly higher than in other groups, while the relative abundance of Basidiomycota, Chytridiomycota, and Mortierellomycota was lower than in other groups. The most frequent fungal genera were *Talaromyces*, *Gliomastix*, and *Russula*, accounting for 0.7–26.5%, 0.08–51.6%, and 0.3–18.9%, respectively, followed by *Sarocladium*, *Fusarium*, and *Cladosporium* (Figure S3b). *Gliomastix* in group AWB had a significantly higher abundance compared with the other four groups, while *Talaromyces* and *Russula* were the dominant genera in groups AA, BA, and BW (Figure 3b). Furthermore, the NMDS results (Figure 4a,b) showed that samples collected from the air (groups AA and BA) were grouped separately from those collected from black spots (group AWB). Figure 4a shows the separation of the bacterial communities between the air and group BW, while no significant differences were observed in fungal communities (Figure 4b). For fungal communities, the samples collected from black spots clustered together and were grouped separately from those collected from the area without macroscopic contamination (group AWN).

### 3.2. Microbial Assembly Processes

**Figure 5:**
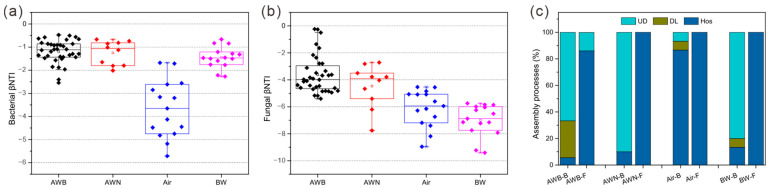
Assembly processes of microbial community. β nearest taxon index (βNTI) in bacteria (**a**) and fungi (**b**) across different groups. Air includes groups AA and BA. Contributions of ecological assembly processes dominating microbial community turnover (**c**). AWB-B, bacteria for group AWB; AWB-F, fungi for group AWB; UD, undominated assembly; DL, dispersal limitation; and Hos, homogeneous selection.

The βNTI results showed that bacterial communities were mainly governed by stochastic processes, while fungal communities were driven by deterministic processes. The βNTI values of bacteria were notably lower compared to fungi, indicating the significance of homogeneous selection (HoS) in influencing fungal communities, while bacterial communities were mainly affected by neutral processes (Figure 5a,b). Undominated assembly (UD) dominated the community assembly process for bacteria in groups AWB, AWN, and BW, accounting for 66.7%, 90%, and 80%, respectively. Bacterial communities were primarily dominated by stochastic processes in air samples (groups AA and BA), where HoS accounted for 86.6%. In fungal communities, HoS was critical for the community assembly in all groups (86.1–100%), and this assembly process in group AWB was weaker compared to other groups. Moreover, dispersal limitation (DL) was present in the bacterial communities of the groups AWB, BW, and air, accounting for 27.8%, 6.7%, and 6.7%, respectively (Figure 5c).

### 3.3. Analysis of Microbial Co-Occurrence Networks

**Figure 6:**
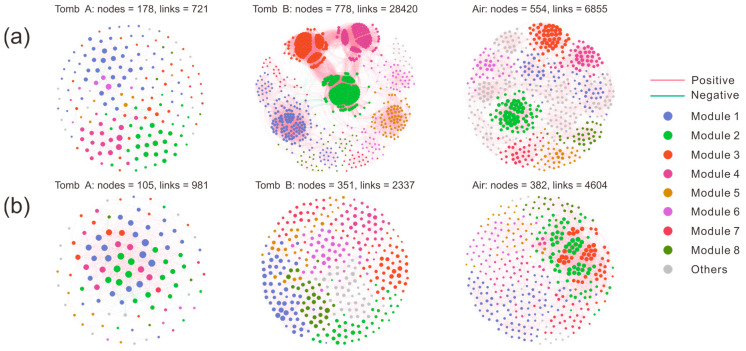
Co-occurrence networks of bacterial (**a**) and fungal (**b**) communities in tomb mural paintings and air. Nodes were colored based on different modules. Connections represent strong (Spearman’s correlation coefficient |r| ≥ 0.8) and significant (*p* < 0.05) correlations. Red and green edges indicate positive and negative interactions, respectively. Tomb A includes groups AWB and AWN; tomb B includes group BW; and air includes groups AA and BA.

Co-occurrence networks were used to estimate the microbial coexistence across the two tombs, via Spearman’s correction analysis with a coefficient (r) > 0.8 and *p*-values < 0.05. The resulting bacterial network of mural paintings from tomb A was composed of 178 nodes associated with 721 edges, in which 80.03% of the links were positively corrected (red edge) and 19.97% were negatively corrected (green edge; Table S2); more nodes and edges were formed in mural paintings of tomb B than in air samples (Figure 6a), indicating that the bacterial communities in tomb B had the most complex network. In fungal communities, air samples exhibited the most complex network with more nodes (382) and edges (4604) than the other two sites (Figure 6b). Similarly to the bacterial network, the enriched ASVs from tomb A formed a simple network with fewer nodes (105), edges (981), and positive connections (91.85%). A set of network topological parameters (e.g., average degree, diameter, and modularity) was used to assess the microbial network complexity, and we found that the topological properties were highly variable among and within diverse environments. The fungal communities in tomb A resulted in a network exhibiting the smallest modularity, average path distance, and higher average clustering coefficient. Airborne bacterial communities had the highest modularity, average path distance, and lower average clustering coefficient. The bacteria–bacteria intra-kingdom network analysis identified the genus *Chiayiivirga* (ASV21798) as a keystone ASV in tomb A, whereas no keystone taxon was found in the fungi–fungi intra-kingdom network (Figure S4).

### 3.4. Potential Functional Pathways in Microbial Communities

**Figure 7:**
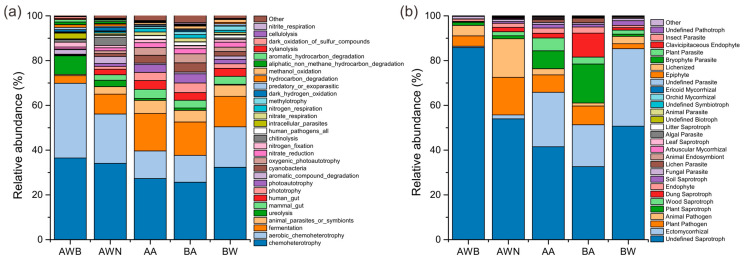
Potential functional pathways in bacterial (**a**) and fungal (**b**) communities.

The FAPROTAX results showed that chemoheterotrophy and aerobic chemoheterotrophy were the predominant functional groups involved in nutrient removal, followed by functions related to fermentation, animal parasites or symbionts, and ureolysis (Figure 7a), suggesting that a large number of microbes gain energy and essential nutrients through the oxidation of organic compounds. Functions assigned to mammal and human guts were significantly richer in air and soil samples, accounting for 8%, 6.9%, 7.1%, and 4.9% in groups AA, BA, BW, and AWN, respectively. Five kinds of functions related to nitrogen metabolism, mainly including nitrate reduction, nitrogen fixation, nitrate respiration, nitrogen respiration, and nitrite respiration, increased significantly in airborne samples. Significant differences in major functional groups among the two groups of samples are shown in Figure S5. Except that intracellular parasites had an extremely significant difference (*p* < 0.001) in the two groups, the differences in other functional groups were statistically significant (*p* < 0.05). There was no significant difference in nitrate reduction, the dark oxidation of sulfur compounds, and nitrous oxide denitrification between groups AWB and AWN. FUNGuild analysis reveals that the Undefined Saprotroph was the predominant fungal ecological guild in the samples from different sites, followed by Ectomycorrhizal, Plant Pathogen, Animal Pathogen, and Plant Saprotroph (Figure 7b). By comparing differences in the functional guilds between groups, we found that the Undefined Saprotroph had the greatest relative abundance in group AWB (85.8%), while the relative abundance of Ectomycorrhizal, Wood Saprotroph, Dung Saprotroph, and Soil Saprotroph in group AWB was lower than in the other four groups. The relative abundances of functional guilds subordinated to Plant Saprotroph and Dung Saprotroph were higher in airborne fungal communities, accounting for an average of 12.7% and 6.4%, respectively. Plant Pathogen and Animal Pathogen in group AWN had a significantly higher abundance compared with group AWB. The relative abundance of Ectomycorrhizal was as high as 34.6% in terms of the functional guilds composition of group BW, which may be attributed to a high abundance of *Russula* [40].

## 4. Discussion

Microbial communities colonizing cultural artifacts is a ubiquitous phenomenon, which may occur during burial, post-excavation, and storage periods [41,42,43]. Among them, fungi represent a major group of organisms that threaten the sustainable conservation of cultural heritage through hyphae penetration, degradative enzymes and pigment secretion, and mediating redox reaction [44,45,46]. In our study, two tombs dating to the late period of the Tang dynasty were excavated (Figure 1b), and a substantial number of exquisite mural paintings were uncovered following the removal of the surface soil layers. After being exposed to the outdoor environment for a period of time, a large number of black spots suspected to be fungal colonies appeared on the mural paintings in the corridor of tomb A. Air and mural painting samples collected from different locations were used to investigate the microbial composition and possible sources of the black spots. For the fungal communities, the black spots are mainly dominated by members of the genera *Gliomastix*, with an average relative abundance of 52.8% in group AWB, followed by unclassified Hypocreales and *Ochroconis*. However, in other areas where there is a less obvious microbial colonization, genera of *Sarocladium*, *Malassezia*, *Fusarium*, and *Plectosphaerella* are the main fungal taxa and only a small proportion of *Gliomastix* was detected (group AWN, Figure S3b). *Gliomastix* appears commonly in soil and has been implicated in the deterioration of mural paintings [47,48]; e.g., the black spots present in murals of the Takamatsuzuka and Kitora Tumuli [49]. Additionally, the genus of *Ochroconis* may contribute to the formation of black spots since the fungal population has been proven to be involved in the formation and wide dissemination of black stains in the Lascaux cave [50,51]. Although the samples in groups AWB, AWN, and BW were all collected from mural paintings, large variations in fungal taxa are observed (Figure 4b), and suggesting that archeological excavations may alter the microbial community structure. Research on airborne microbes contributes to our understanding of the sources and transmission pathways of key microbial taxa [52,53], providing insight into the potential threat to cultural heritage [54] and public health. For instance, fungi produce harmful substances that seriously threaten the occupational health of heritage conservators and restorers [55]. Although the samples of groups AA and BA were collected from different sampling sites, there was no statistically significant difference between the two groups in species richness and diversity (Figure 2), and this result was further supported by NMDS analyses (Figure 4), which may be attributed to the open-air environment of the tombs. Air samples were grouped separately from those collected from black spots, and the dominant microbial species in group AWB were rarely detected in groups AA and BA (Figures S2 and S3), indicating less possibility of the dissemination of melanized fungal spores through air. Conversely, no significant differences in the microbial community structure are detected between mural paintings in tomb B and the air, especially in fungal communities (Figure 4b), suggesting the possibility of microbial dispersal and migration events. The microbial profiling of mural paintings in tomb B was conducted immediately following the removal of the surface soil layers, and the detected surface microbiota predominantly reflects soil-derived microbial populations. Meanwhile, as a recognized reservoir of microorganisms in terrestrial ecosystems [56], soil contributes significantly to microbial diversity and dispersal [57], which may help explain why group BW and the air have a similar pattern in the microbial community. For the bacterial communities, the genera of *Promicromonospora*, *Phyllobacterium*, *Dyadobacter*, *Singulisphaera*, *Chitinophaga*, and *Olivibacter* are mainly detected in the black-spots-contaminated area, whereas *Bacteroides*, *Alistipes*, and *Akkermansia* prevail in uncontaminated zones and air. The bacterial genus *Pseudonocardia* within the phylum Actinobacteria is also common on rocky substrates of subterranean environments [58], and has been detected widely in mural paintings and air (Figure S2), indicating a potential threat to these precious painted artifacts.

Clarifying the microbial dynamics and assembly processes is critical for predicting the response of microbial populations to environmental disturbance [59]. Classic ecological models have been used to interpret microbial assembly mechanisms and microbial interactions within the deteriorated limestone, revealing stability in ecosystem function and non-random community assembly [60]. Microbial assembly processes in the Lascaux cave suggest that always-rare microbial taxa involved in the biodegradation of the chemical biocides play a significant role in ecological resilience [31]. In this study, deterministic processes (homogeneous selection) dominate the airborne microbial assembly, in accordance with the views that stronger deterministic processes might be linked to a less abundant resource supply [61]. Homogeneous selection is usually determined by a similar selection pressure imposed by environmental conditions, thereby leading to much less variation in microbial community structure. Fungal communities in mural paintings are formed primarily by deterministic processes, contrary to the pattern observed in bacterial communities (Figure 5). A general relationship between organism body size and the microbial community assembly indicates that smaller bacteria are relatively more influenced by dispersal-based stochastic processes, while deterministic processes significantly influence fungal community assembly [62]. Bacterial community construction tends to be based on undominated processes, and the relative contributions of these processes differ among the contaminated and uncontaminated surfaces, highlighting the complexity of community assembly.

A complex microbial network system uncovers the possible ecological connections and determines soil ecosystem functions [63]. Fluctuations in environmental conditions also change the co-occurrence patterns of microbial communities, thereby influencing network complexity and the stability of microbes [64]. In this study, compared to mural paintings covered with soil, microbial communities in uncovered surfaces display much less network complexity, which is reflected by the decreasing number of nodes and links (Figure 6). The microclimate in the burial environment is critical for maintaining microbial community structure stability and the historical artifacts’ sustainability [65]. The soil provides an ecological niche where microbial populations construct a compact and stable co-occurrence network before an intact tomb is excavated, whereas the original microbial community balance may be disrupted once the soil layer is removed. Climatic variables, including temperature, humidity, light, etc., not only affect the complexity and distribution of soil microbial networks, but also alter ecosystem functions of the active microbial community. The open tomb environment increases CO_2_ and light levels, which in turn results in the enrichment of phototrophic microorganisms and accelerates carbon cycling. Microbes colonizing the corridor of the tomb exhibit lower levels of phototrophic bacteria and functions, which may be attributed to insufficient lighting (Table S1).

Numerous studies have indicated that biogeochemical cycles are largely driven by microbial activity, and these processes pose a potential threat to calcareous stone monuments [46,66]. Importantly, different groups of microorganisms involved in nitrogen and sulfur cycles are capable of secreting acidic chemicals that dissolve carbonate minerals, thereby accelerating stone deterioration. FAPROTAX results suggest that the microbial functional profiles involved in nitrogen cycling are enriched in air and mural paintings, potentially damaging these painted artifacts through the corrosion reaction between organic acids and lime or pigments. Additionally, microbial communities in contaminated areas have functions in aromatic and aliphatic hydrocarbon degradation, which pose a threat to the stability of protective organic coatings. Saprotrophic fungi play a major role in the nutrient cycling by enabling the decomposition of organic matter into substances that are then absorbed by the cells to feed the organism [67]. Ectomycorrhizal fungi are capable of secreting organic acids that enable ectomycorrhizal plants to utilize essential nutrients directly from minerals, playing key roles in forest ecosystems [68]. *Russula* is one of the most important ectomycorrhizal genera, with hundreds of species that are globally distributed [69]. *Russula* accounts for a much higher proportion of the fungal communities in air than in contaminated mural paintings, which is consistent with the above-mentioned conclusion that airborne fungi are mostly derived from soil. In addition, it is worth noting that animal pathogenic fungi, such as *Malassezia globose* [70] and *Fusarium* [71], are detected in the majority of the samples, emerging as a threat to human and animal health. BugBase functional analysis (Figure S6) confirms that potential pathogenic phenotypes of bacterial populations are found to be highly enriched in air and mural paintings (with an average of 16.4% per group), which serve as a warning that cultural heritage microbiome research should also pay attention to personal and public safety issues. Meanwhile, the pathogenic-related signatures and resistome profiles within the microbiome in the burial environment represent a potential health threat to surrounding populations [43,72,73]. In subsequent studies, long-term monitoring will be performed to assess the dynamic changes in microbial communities and their responses to environmental changes. Additionally, culture-independent methods will be used to evaluate the potential threat of microbes to mural paintings and public health.

## 5. Conclusions

In summary, the distribution of the microbial communities residing in the tomb mural paintings and their surrounding air was studied, and the composition of the core microorganisms causing black spot contamination was also identified. The core microbial taxa, mainly including *Gliomastix* and *Ochroconis*, were highly abundant in group AWB and rarely detected in the air and uncontaminated mural paintings. The air samples within the two tombs were closely clustered but were grouped separately from those collected from black spots, suggesting less possibility of the dissemination of melanized fungal spores through air. No significant differences in microbial community structure were observed between group BW and the air, implying potential microbial dispersal events between the soil and air. Archeological excavations not only affect microbial assembly processes but also change the co-occurrence patterns of microbial communities. Functional predictions reveal the putative presence of microbial functional profiles involved in nitrogen cycling, hydrocarbon degradation, and animal and human pathogens, posing a potential threat to the sustainable preservation of cultural relics and public health. Taken together, this study provides new insights into the changes in the microbial community structure of ancient tomb mural paintings before and after archeological excavations.

## Data Availability

The original contributions presented in the study are included in the article material, further inquiries can be directed to the corresponding author.

## Supplementary Materials

The following supporting information can be downloaded at www.mdpi.com/article/10.3390/microorganisms13040755/s1: Figure S1: The distribution of mural painting and air samples in the two tombs; Figure S2: Relative abundance of the top 15 species at the bacterial phylum level (a) and genus level (b); Figure S3: Relative abundance of the top 15 species at the fungal phylum level (a) and genus level (b); Figure S4: The distribution of keystone taxa in bacterial (A) and fungal (B) communities; Figure S5: Significant differences in major functional group between groups AWB and AWN; Figure S6: BugBase functional analysis of bacterial community in mural paintings and air; Table S1: Data of microclimate parameters; Table S2: Network Topology Indices.
