# Supplementary material for: Uncovering Microbial Diversity and Community Structure of Black Spots Residing in Tomb Mural Painting
Source: Microorganisms. 2025 Mar 26;13(4):755. doi: 10.3390/microorganisms13040755 (PMC12029219; doi:10.3390/microorganisms13040755)
Supplement: Supplementary file 1 [file microorganisms-13-00755-s001.zip › microorganisms-3519744-supplementary.pdf]

microorganisms MDPI

Supplementary Materials

Uncovering Microbial Diversity and Community Structure of Black Spots Residing in Tomb Mural Painting

Qiang Li [1,†], Zhang He [2,†], Zeng Wang [2], Aidong Chen [2] and Chao Wu [3,*]

[1] School of Art and Archaeology, Zhejiang University, Hangzhou 310028, China
[2] Shaanxi Provincial Institute of Archaeology, Xi'an 710054, China
[3] School of Humanities, Zhejiang University, Hangzhou 310058, China
* Correspondence: wu_chao@zju.edu.cn
† These authors contributed equally to this work.

Table S1. Data of microclimate parameters

			Temperature	Humidity	Illuminance level	CO_2 concentration
Tomb A	AWB	AW1	18.0	81.7	11.8	522
		AW21	18.0	82.3	< 5.0	518
		AW22	17.9	83.2	< 5.0	522
		AW31	18.0	83.0	< 5.0	524
		AW32	17.9	82.2	< 5.0	527
		AW33	17.9	81.6	< 5.0	598
		AW41	18.0	81.6	< 5.0	494
		AW42	18.0	85.0	< 5.0	494
		AW5	17.9	83.7	< 5.0	457
	AWN	AW61	17.9	82.2	< 5.0	538
		AW62	17.9	83.0	< 5.0	620
		AW71	18.3	81.9	65.2	535
		AW72	18.2	83.2	68.3	532
		AW73	18.0	84.3	19.1	555
	AA	AA12	18.1	82.3	68.0	532
		AA13	18.0	84.1	18.0	555
		AA2	17.9	85.0	< 5.0	538
		AA3	18.0	82.0	< 5.0	522
Tomb B	BA	BA1	18.6	80.6	800	500
		BA2	18.5	76.4	150	454
	BW	BW1	18.9	77.6	753.7	536
		BW2	18.9	77.6	753.7	532
		BW3	18.5	80.6	225	536
		BW4	18.7	80.6	225	471
		BW5	18.9	77.6	730	471
		BW6	18.6	78	730	454

Table S2. Network Topology Indices

	Network Topology Indices	tomb A	tomb B	Air
Bacteria	Total nodes	178	778	554
	Total links	721	28420	6855
	Positive connections	80.03%	99.81%	100%
	Average degree	8.10	73.059	24.747
	Diameter	10	9	8
	Modularity	0.639	0.731	0.805
	Average clustering coefficient	0.487	0.722	0.616
	Average path distance	3.874	3.307	4.026
	Density (D)	0.046	0.094	0.045
Fungi	Total nodes	105	351	382
	Total links	981	2337	4604
	Positive connections	91.85%	99.91%	99.04%
	Average degree	18.686	13.316	24.105
	Diameter	8	8	8
	Modularity	0.259	0.695	0.546
	Average clustering coefficient	0.624	0.495	0.528
	Average path distance	2.472	4.016	3.424
	Density (D)	0.18	0.038	0.063

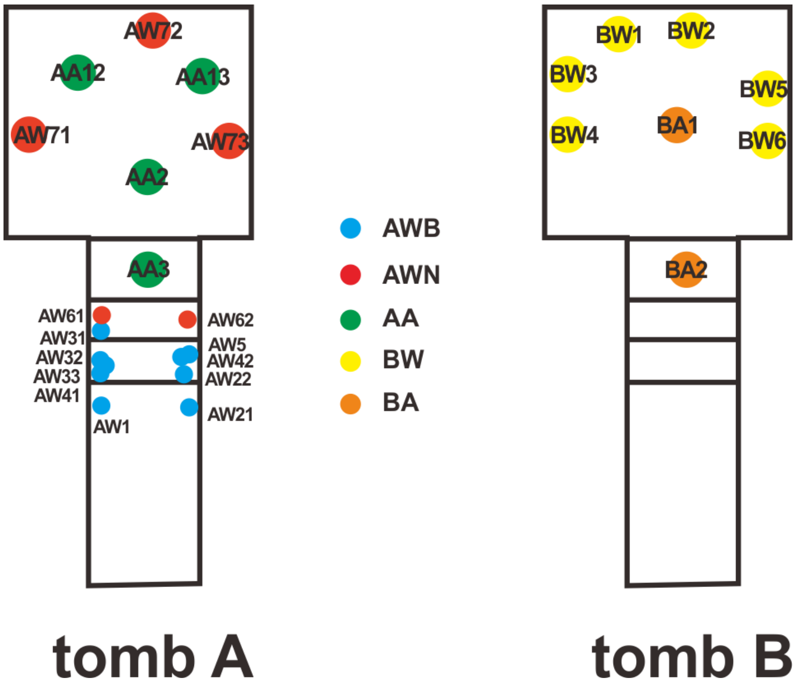

Figure S1. The distribution of mural painting and air samples in the two tombs.

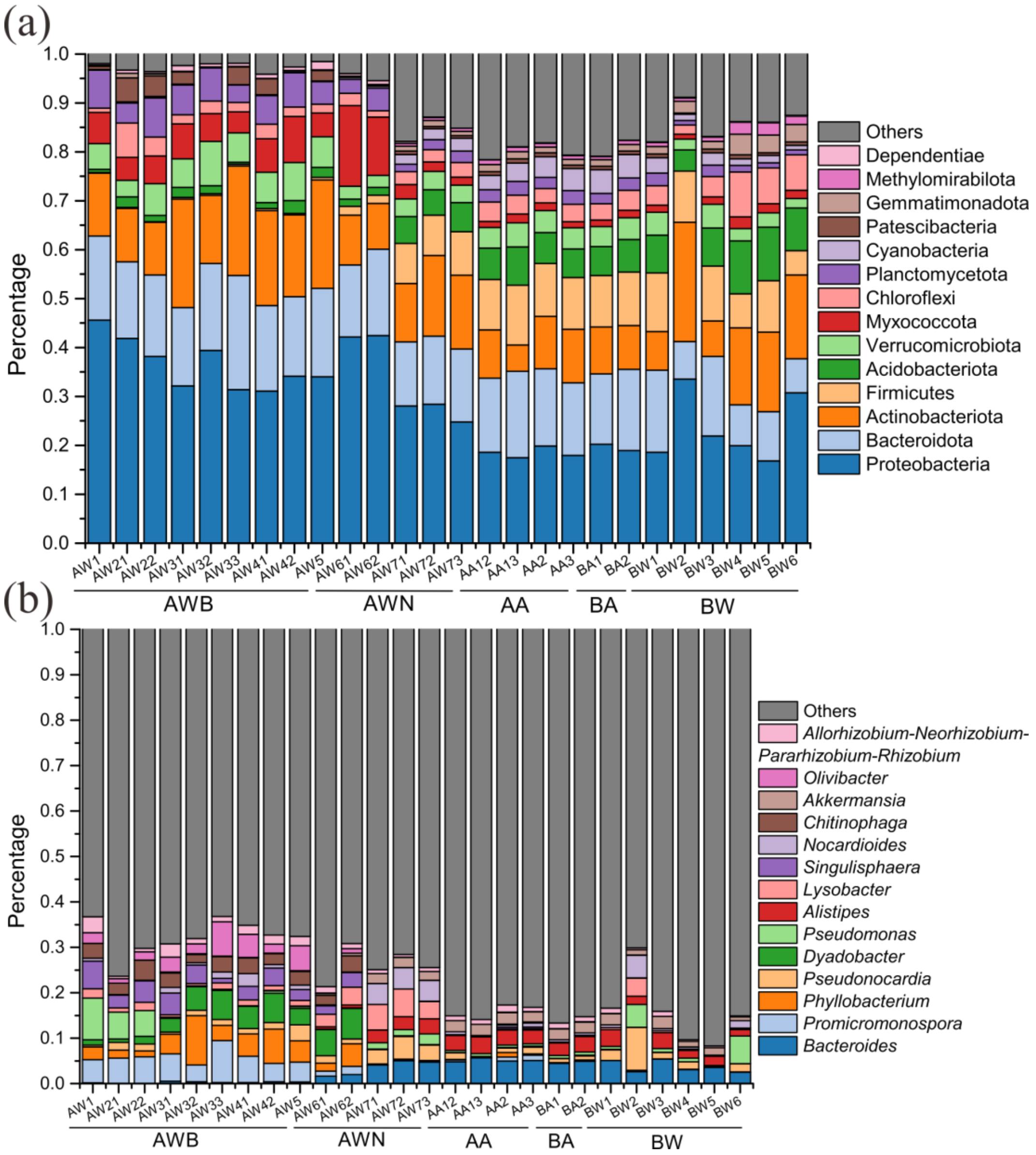

Figure S2. Relative abundance of the top 15 species at the bacterial phylum level (a) and genus level (b).

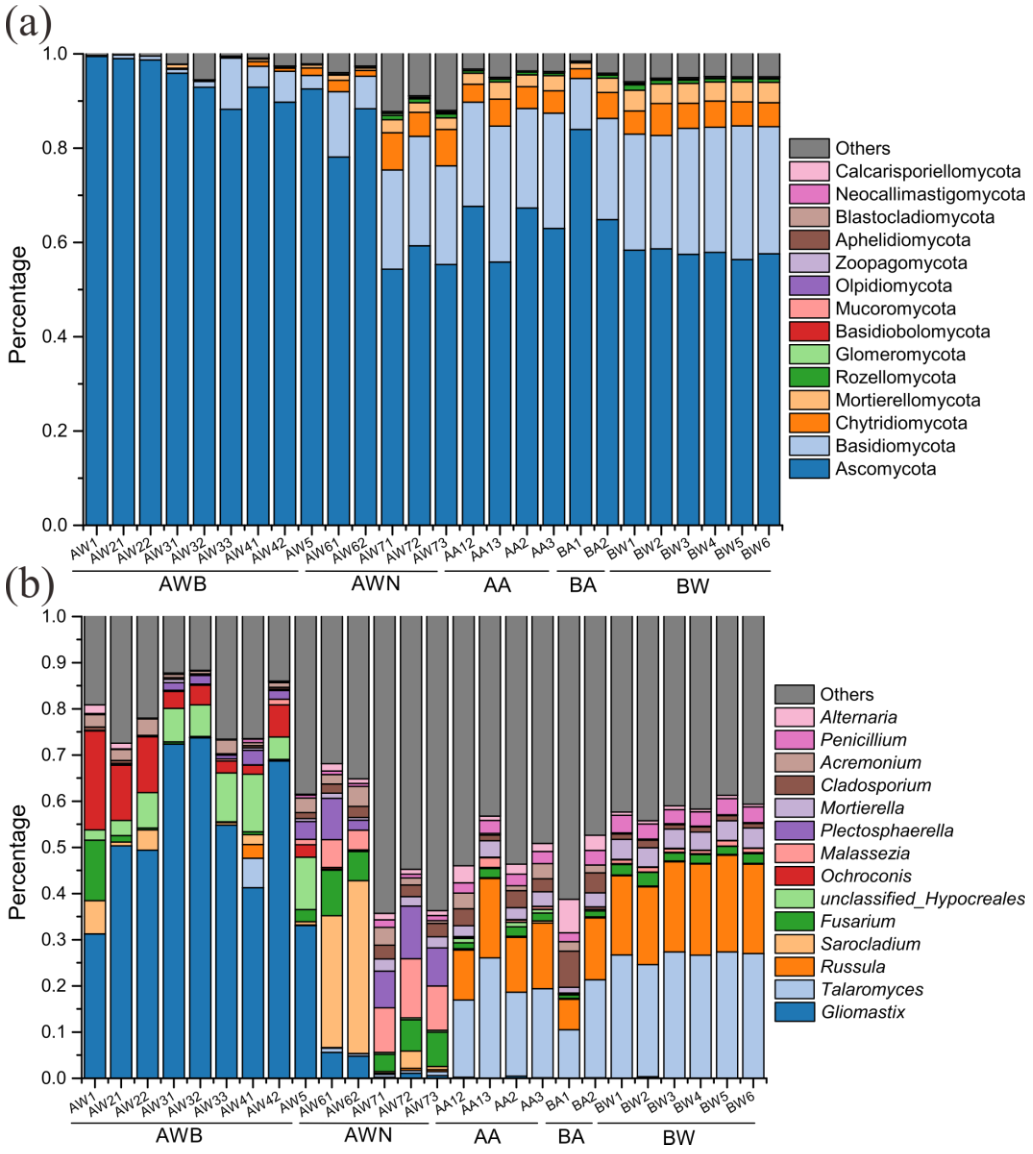

Figure S3. Relative abundance of the top 15 species at the fungal phylum level (a) and genus level (b).

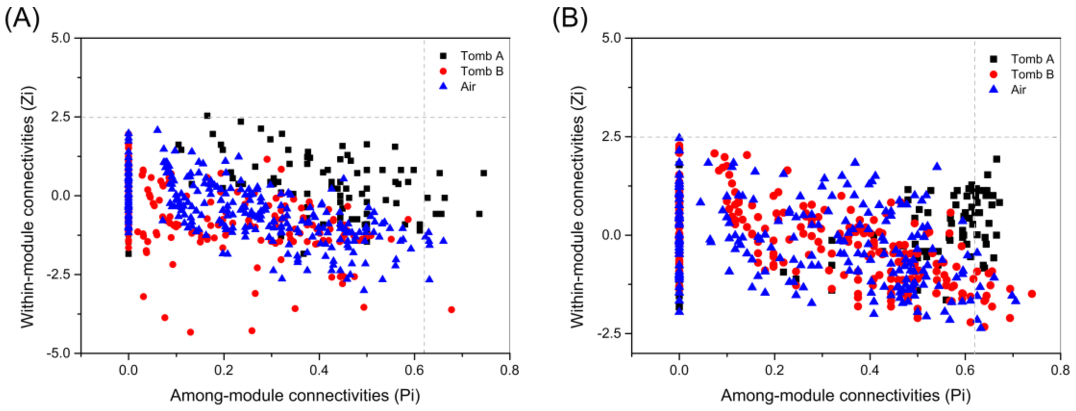

Figure S4. The distribution of keystone taxa in bacterial (A) and fungal (B) communities.

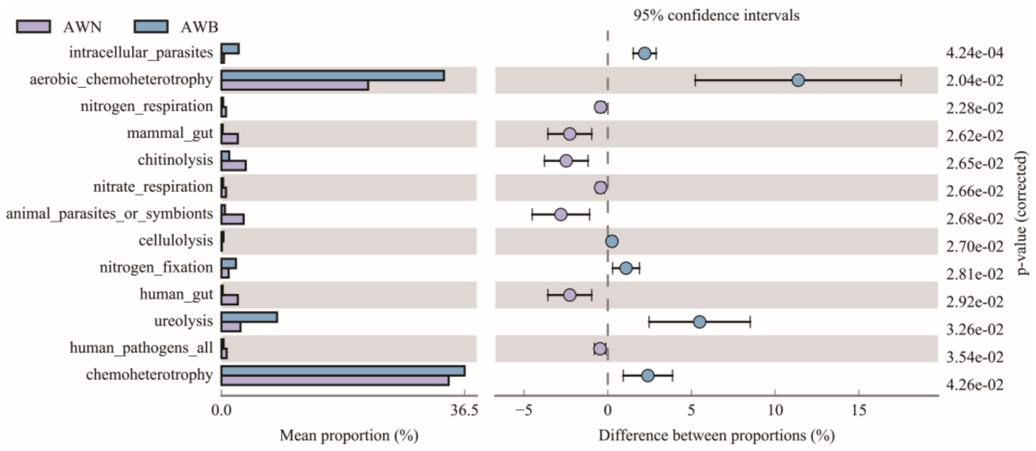

Figure S5. Significant differences in major functional group between groups AWB and AWN.

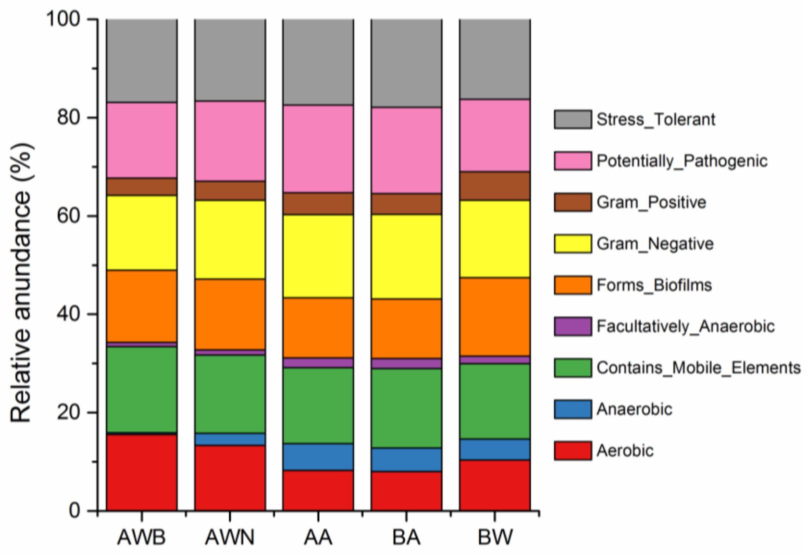

Figure S6. BugBase functional analysis of bacterial community in mural paintings and air.
